# Effect of APOE ε4 on multimodal brain connectomic traits: a persistent homology study

**DOI:** 10.1186/s12859-020-03877-9

**Published:** 2020-12-28

**Authors:** Jin Li, Chenyuan Bian, Dandan Chen, Xianglian Meng, Haoran Luo, Hong Liang, Li Shen, for the Alzheimer’s Disease Neuroimaging Initiative

**Affiliations:** 1 College of Automation, Harbin Engineering University, 145 Nantong Street, Harbin, 150001 Heilongjiang China; 2 School of Computer Information and Engineering, Changzhou Institute of Technology, Changzhou, 213032 China; 3 Department of Biostatistics, Epidemiology and Informatics, Perelman School of Medicine, University of Pennsylvania, B306 Richards Building, 3700 Hamilton Walk, Philadelphia, PA 19104 USA

**Keywords:** APOE ε4, Brain network, Persistent homology, Alzheimer’s disease

## Abstract

**Background:**

Although genetic risk factors and network-level neuroimaging abnormalities have shown effects on cognitive performance and brain atrophy in Alzheimer’s disease (AD), little is understood about how apolipoprotein E (APOE) ε4 allele, the best-known genetic risk for AD, affect brain connectivity before the onset of symptomatic AD. This study aims to investigate APOE ε4 effects on brain connectivity from the perspective of multimodal connectome.

**Results:**

Here, we propose a novel multimodal brain network modeling framework and a network quantification method based on persistent homology for identifying APOE ε4-related network differences. Specifically, we employ sparse representation to integrate multimodal brain network information derived from both the resting state functional magnetic resonance imaging (rs-fMRI) data and the diffusion-weighted magnetic resonance imaging (dw-MRI) data. Moreover, persistent homology is proposed to avoid the ad hoc selection of a specific regularization parameter and to capture valuable brain connectivity patterns from the topological perspective. The experimental results demonstrate that our method outperforms the competing methods, and reasonably yields connectomic patterns specific to APOE ε4 carriers and non-carriers.

**Conclusions:**

We have proposed a multimodal framework that integrates structural and functional connectivity information for constructing a fused brain network with greater discriminative power. Using persistent homology to extract topological features from the fused brain network, our method can effectively identify APOE ε4-related brain connectomic biomarkers.

## Background

Alzheimer’s disease (AD) is a chronic neurodegenerative brain disease that gradually causes cognitive deterioration [1, 2]. Although studies on the specificity of disease stage is underway to identify potential biomarkers, early diagnosis of vulnerability to AD—prior to the onset of clear cognitive symptoms—is still challenging [3, 4]. In past years, neuroimaging-based techniques have been used to reveal the neuronal interaction patterns of anatomically segregated brain regions in AD via constructing brain connectome [1, 5]. However, traditional network-level neuroimaging approaches have limited ability to discriminating normal aging from early AD [6]. Neuroimaging genetics offers a promising strategy for detecting potential early biomarkers of AD and improving the understanding of neurobiological features associated with genetic polymorphisms at risk for AD [7]. In particular, apolipoprotein E (APOE) ε4 allele is the uppermost genetic risk factor for developing sporadic AD, which is observed in up to 50% of all AD cases [8]. We propose here a brain imaging genetics study to examine the effect of the APOE ε4 genotype on brain connectomic traits. Our goal is to quantify the functional and structural brain differences from the perspective of genetics in patients, who may be undergoing early neuropathological changes in the pathological cascade leading to disease. Currently, an ample number of studies [9, 10] have investigated the brain connectivity features of APOE ε4 carriers. These existing studies found specific and consistent alterations in brain network, especially involving decreased functional connectivity within default mode network (DMN). Particularly, most of these approaches for characterizing APOE-related network differences are based on pairwise correlation such as Pearson’s correlation. Nevertheless, some studies [11, 12] have demonstrated that the neurological processes involve the interactions of many co-activated brain regions (i.e., more than two brain regions) rather than just the pairwise variant-trait associations.

To address this problem, the least absolute shrinkage and selection operator (Lasso) and sparse representation have been applied to construct a sparse brain network by considering more complex interactions among multiple co-activated brain regions [13]. However, the Lasso approaches have their own limitations. For example, most of them use a fixed regularization parameter *λ* that may not be optimal to control the model sparsity, which can lead to an uncertainty to quantify the sparse brain networks [14]. Moreover, another problem with Lasso is, feature extraction of sparse network needs a constructed network with precise connection strengths [15]. However, traditional Lasso method has been shown biased, and may not provide reliable estimation for building brain networks. Therefore, a subsequent connectivity strength estimation process should be performed to eliminate the shrinking effect, which naturally adds the complexity of modeling. In order to address the above limitations, a persistent homology (PH) [16–19] method is newly proposed in this work. Our novel method constructs the brain network over multiscale regularization parameter space and only focuses on the network structure (binary network) rather than connection strength (weight network) between regions. Hence, we hypothesize that the combination of PH and SR may yield a potential path to identify more sensitive brain network-level biomarkers.

Currently, a lot of brain network modeling methods only consider the neurological processes from a single modality [20], while compelling evidences have demonstrated the benefit of acquiring and fusing complementary information via different neuroimaging modalities for accurate classification [21, 22]. Specifically, diffusion-weighted MRI (dw-MRI) has been applied to map white matter tractography that outputs structural connectivity (SC). On the other hand, resting state functional MRI (rs-fMRI) measures intrinsic functional connectivity (FC) through spontaneous fluctuations of brain activity. Joint investigation of dw-MRI and rs-fMRI data can offer a complete characterization of the brain network incorporating both structural and functional connectivity. For example, Qi et al. [23] proposed a framework for integrating the multimodal imaging data of diffusion-MRI and fMRI. Their results suggested that the multimodal fusion can effectively detect potential imaging biomarkers of working memory deficits in schizophrenia. Korthauer et al. [6] integrated rs-fMRI and dw-MRI data in a single network for investigating a functional-structural network difference in apolipoprotein E (APOE) *ε*4 carriers and non-carriers. In their study, integrating multiple neuroimaging modalities was demonstrated to be a more effective method to detect network-level biomarkers compared to conventional single modality method. Based on these findings, we hypothesize that a multimodal fusion method may further improves the statistical performance between APOE ε4 carriers and non-carriers.

In this paper, we focus on identifying APOE ε4 related differences from the perspective of brain connectome. The main methodological contributions are threefold. First, we propose a novel multimodal brain network modeling method for detecting the differences of APOE ε4-associated brain connectivity. Our method integrates the multimodal information from both rs-fMRI and dw-MRI. Specifically, a generalized fused Lasso method is applied to linearly regress rs-fMRI signals (BOLD time series), and is guided by SC prior information. Second, we develop a multiscale network quantification method using PH for the proposed model. We show that after integrating the brain network information with different sparsity for each subject, PH can effectively characterize the multiscale networks via graph filtration, which overcomes the uncertainty of optimal parameter selection. To the best of our knowledge, no previous methods ever fused both multimodal brain modeling and PH into a sparse representation, upon which our novel framework is built. Third, we design a connectivity pattern identification method based on PH features. Different from the existing methods, our method can characterize the APOE ε4-related specific loop structures in the brain network, which can reflect meaningful biological communication patterns. Finally, we perform our experimental study using rs-fMRI and dw-MRI data from the publicly available Alzheimer's Disease Neuroimaging Initiative (ADNI) database. We demonstrate the promise of our method over the competing methods on both statistical performance and connectivity pattern identification.

## Methods

### Participants

Data used in the preparation of this article were obtained from the ADNI database (adni.loni.usc.edu). In this study, APOE genotype, rs-fMRI, dw-MRI, and T1 imaging data were collected from 63 subjects, and divided into two groups: APOE ε4 carriers (N = 27, 17 males and 10 females, age 63–89) and APOE ε4 non-carriers (N = 36, 20 males and 16 females, age 61–87).

### Genotype and neuroimaging data

For genotyping, subjects carrying at least one APOE ε4 allele were defined as APOE ε4 carriers (genotype ε4/ε4 and ε4/ε3), while subjects with the genotype ε3/ε3 were classified as APOE ε4 non-carriers. Subjects with the ε2 allele (including genotypes ε2/ε2, ε2/ε3, and ε2/ε4) were excluded in this study. For neuroimaging data, all MRI data were acquired with a Siemens 3 T scanner with the following parameters: (1) rs-fMRI data involved that TE (echo time) = 30 ms, TR (repetition time) = 3000 s, FA (filp angle) = 90 degree, slice thickness = 3.4 mm, the number of slices = 197; (2) dw-MRI data were acquired with gradient directions = 54, TE = 56 ms, TR = 7200 ms, voxel size = 2 × 2 × 2mm^3^, FA = 90 degree; (3) T1 image data were acquired with FA = 9 degree, acquisition plane = SAGITTAL, slice thickness = 1.2 mm, TE = 2.95 ms, T1 = 900 ms, TR = 2300 ms.

### Data preprocessing

T1 is a structural MRI (sMRI) modality capturing brain morphometry. T1 is often used as a reference image to which dw-MRI and rs-fMRI data can be registered. After that, all three modalities are aligned to the same reference so that multimodal data analysis can be facilitated.

For rs-fMRI data, we used SPM8 (https://www.fil.ion.ucl.ac.uk/spm/software/spm8/) and DPABI [24] for preprocessing as follows: removing the first 10 time points; slice timing correction; spatial correction for head motion; co-registering the individual T1 image to the mean functional image after realignment by using DARTEL (a fast diffeomorphic anatomical registration algorithm to calculate the transformations from individual native space to MNI space); smoothing using Gaussian kernel with FWHM (full-width-athalf-maximum) of 4 × 4 × 4mm^3^; standardization to reduce the impact of nuisance covariates including head motion parameters, white matter signal and cerebrospinal fluid signal. For quality control, the rs-fMRI data with greater 2.5 mm and 2.5 degree in max head motion are excluded.

For dw-MRI data, we used a package called pipeline toolbox for analyzing brain diffusion images (PANDA) [25] developed based on the FMRIB Software Library (FSL, https://fsl.fmrib.ox.ac.uk/fsl/). Specifically, it includes estimating a brain mask using *bet* command of FSL based on b_0_ image without diffusion weighting; removing the non-brain spaces by *fslroi* command; eddy-current correction; calculation for diffusion tensor metrics by *dtifit* command; deterministic white matter tractography within brain using *dti_recon* and *dti_tracker* commands of the Diffusion Toolkit (http://trackvis.org/dtk/). We found that the *bet* command of FSL shows better results for brain tissue extraction than PANDA, so T1 image without skull is achieved using this approach. Each subject’s FA image is co-registered to its corresponding T1 image based on *flflirt* in FSL for defining network nodes. For quality control, the dw-MRI data with significant distortion in co-registration with FA and T1 image or with T1 image and MNI template were excluded from the study. Of note, since T1 scans were used for jointly guiding both dw-MRI and rs-fMRI registrations, the multimodal images were registered onto a same reference template.

### Multimodal brain network

**Fig. 1:**
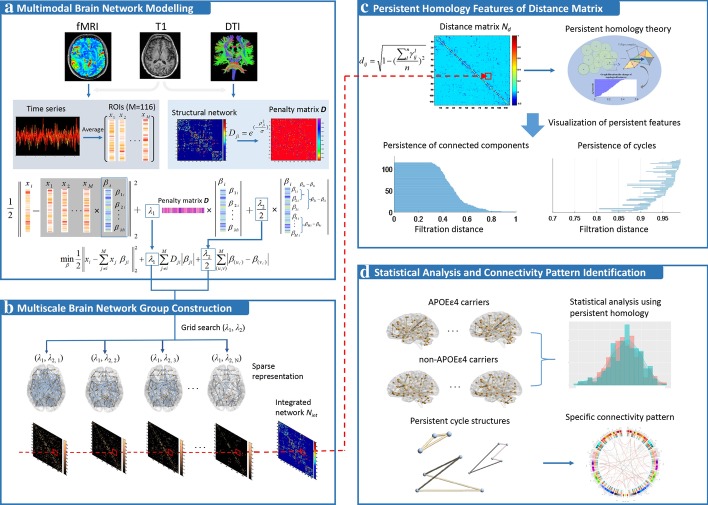
**a** The proposed multimodal framework, where dw-MRI network as a constraint is used to guide the regression of rs-fMRI BOLD time series to create fused brain connectivity network; **b** a sequence of brain networks with different sparsity are constructed to form integrated networks, which are prepared for subsequent graph filtration analysis to extract persistent homology measures; **c** each integrated network is converted to distance network, and persistent homology is applied to quantify the distance network by evaluating the persistence of connected components (PH-0) and cycles (PH-1); **d** statistical analysis is performed to capture the difference between APOE ε4 carriers and non-carriers. Also, we extract specific loop structures by identifying connectivity patterns using PH-1

The framework of multimodal brain network modeling is shown in Fig. 1a. Let us assume that we have **N** subjects and **M** regions of interest (ROIs). For each ROI, a regional mean fMRI BOLD time series is available. We suppose that the BOLD time series with respect to the *i-*th ROI can be denoted as $x_{i} = \{ x_{1i} ,x_{2i} ,\ldots,x_{Ti} \} \in R^{T}$, where **T** is the number of time points (we have 280 time points). $\beta_{i} = \{ \beta_{1i} ,\beta_{2i} ,\ldots,\beta_{Mi} \} \in R^{M}$ is the coefficient vector that represents the indices of other co-activated ROIs associated with the *i-*th ROI. We can estimate the whole-brain network ${\varvec{B}} = \{ {\varvec{\beta}}_{1} ,{\varvec{\beta}}_{2} ,\ldots,{\varvec{\beta}}_{M} \} \in R^{M \times M}$ by solving the following *l*_1_-norm regularization problem:

1 $$\mathop {\min }\limits_{\beta } \frac{1}{2}\left\| {x_{i} - \sum\limits_{j \ne i}^{M} {x_{j} \, \beta_{ji} \, } } \right\|_{2}^{2} + \, \lambda_{1} \, \sum\limits_{j \ne i}^{M} {\left| {\beta_{ji} } \right|}$$

where *λ*_1_ is a non-negative regularization parameter controlling the sparsity of the brain network. A larger *λ*_1_ forces more coefficients to be zeros, i.e., more values in coefficient vector $\{ {\varvec{\beta}}_{i} \}_{i = 1}^{M}$ equal to zero, which is able to select the strongly co-activated ROIs from the *i-*th ROI to the other ROIs. In general, functional network estimated by rs-fMRI can measure the temporal correlation of anatomically segregated brain regions, while a structural network based on dw-MRI is formed by characterizing the white matter fiber tracts. In order to integrate these two sources of complementary information, we propose to incorporate the structural network into Eq. (1) to guide modeling of the functional brain network. Thus, a multimodal network construction can be formulated as:

2 $$\mathop {\min }\limits_{\beta } \frac{1}{2}\left\| {x_{i} - \sum\limits_{j \ne i}^{M} {x_{j} \, \beta_{ji} \, } } \right\|_{2}^{2} + \, \lambda_{1} \, \sum\limits_{j \ne i}^{M} {D_{ji} \left| {\beta_{ji} } \right|}$$

where *D*_*ji*_ represents the structural connectivity information computed from the dw-MRI data. The neurological basis of *D*_*ji*_ is that the coupling of functional and structural connectivity can be regarded as significantly correlated with brain development. A stronger FC is likely to be attributed to a larger SC, and in turn a lower penalty to $\{ {\varvec{\beta}}_{i} \}_{i = 1}^{M}$. Therefore, we define the *D*_*ji*_ as an inverse proportion function of SC. In particular, we set $D_{ji} = \exp ( - \rho_{ji}^{2} /\sigma )$ to penalize the estimated connection between the *j-*th and *i-*th ROIs, where *ρ*_*ji*_ denotes elements in the structural brain network, and *σ* is the average of standard variances of all subjects’ structural network elements (i.e., *ρ*_*ji*_).

Note that the aforementioned model only characterizes the extent of the influence from the *i-*th ROI to other ROIs, ignoring the temporal dependency among the other ROIs. Thus, we further introduce an additional source of guidance, named generalized fused Lasso, to pursue smoothness between the pairwise ROIs as follows:

3 $$\mathop {\min }\limits_{\beta } \frac{1}{2}\left\| {x_{i} - \sum\limits_{j \ne i}^{M} {x_{j} \, \beta_{ji} \, } } \right\|_{2}^{2} + \, \lambda_{1} \, \sum\limits_{j \ne i}^{M} {D_{ji} \left| {\beta_{ji} } \right|} + \frac{{\lambda_{2} }}{2} \, \sum\limits_{(u,v)}^{M} {\left| {\beta_{(u, \cdot )} - \beta_{(v, \cdot )} } \right|}$$

where $\frac{{\lambda_{2} }}{2} \, \sum\nolimits_{(u,v)}^{M} {\left| {\beta_{(u, \cdot )} - \beta_{(v, \cdot )} } \right|}$ is the generalized fused Lasso term, which is used to adaptively control the similarity by shrinking the difference between ROIs toward zero. Moreover, *l*_1_-norm regularization is used to penalize the fusion term, which results in a sparse pattern.

### Persistent homology quantification

**Fig. 2:**
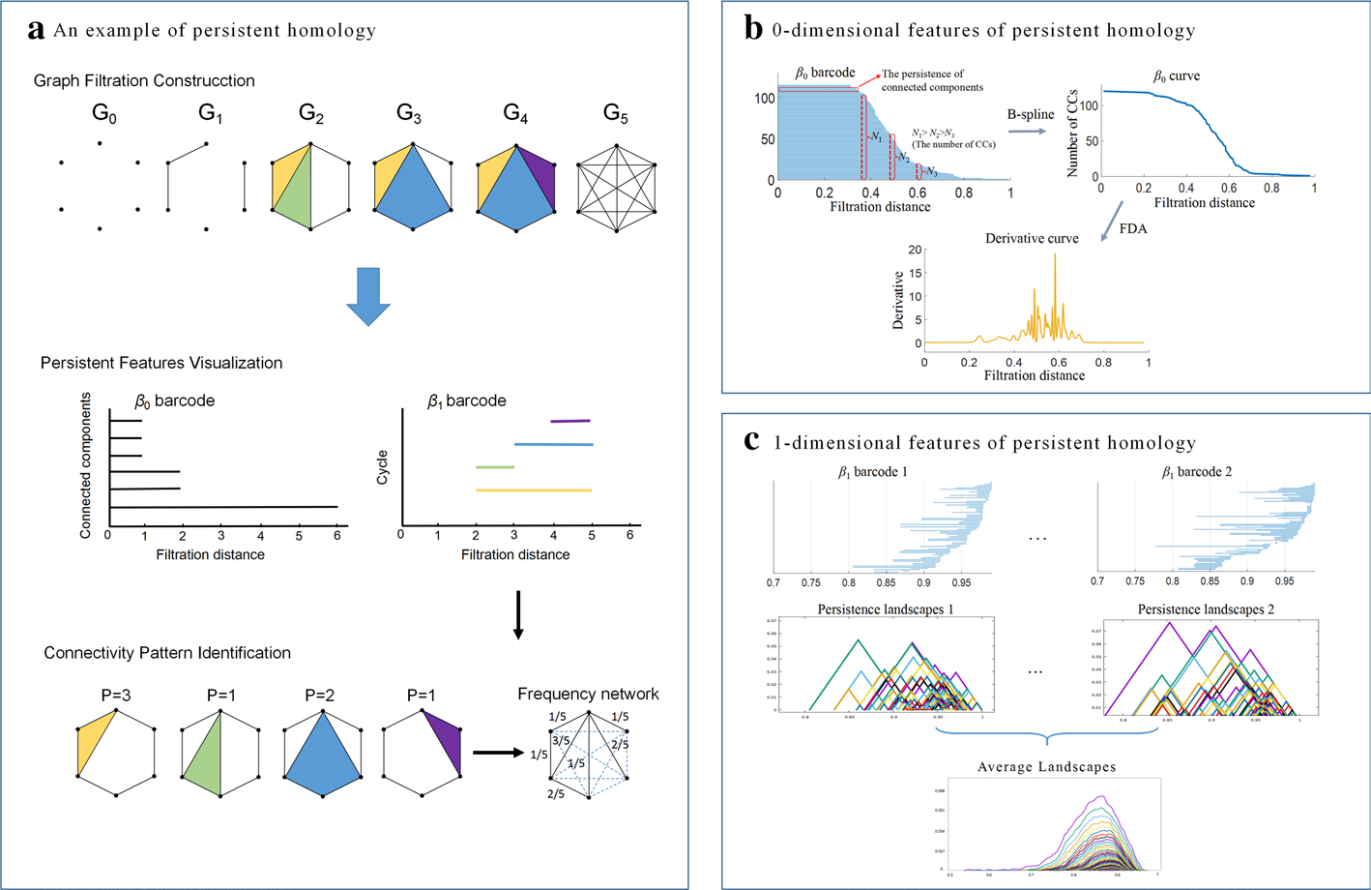
**a** The flowchart of constructing graph filtration, and persistent features in graph filtration can be visualized using barcode. For *β*_0_ barcode, there are 6 connected components in filtration distance 0, which are corresponding to 6 bars. After integrating two connected components into one unit, one of the bars that represents the original components disappears; the other as the new connected component extends to next step. Therefore, the bars in step 1 become 3 that are corresponding to 3 connected components in G_1._ For an example of *β*_1_ barcode, we observe that the cycle with yellow is born in filtration step 2, and continues to step 4 before it is completely covered in filtration step 5. Subsequently, frequency network can be obtained by integrating all cycle structures into a network, where *P* is the persistent interval, the values in frequency network represent probability-of-appearance of an edge. **b** We use functional data analysis (FDA) to fit *β*_0_ barcode to *β*_0_ curve, and further calculate the corresponding derivative curve. **c** Persistence landscapes is applied to convert *β*_1_ barcode to landscape layers; furthermore, we can compute the unique average for a collection of persistent landscapes

*Persistent homology* (PH)—a mathematical formalism from computational topology [26]—can explore the persistence of topological invariants in a network, including connected components, cycles, voids, etc. More specifically, a process called *graph filtration* generates a series of nested simplicial complexes by varying the value of a filtration parameter [27]. Furthermore, we can track the persistence over the graph filtration from the formation (birth) of a topological feature until it disappears (death) by being connected to a neighboring network (Fig. 2a). In general, we define *0-dimensional features* (PH-0) as the persistence of connected components, *1-dimensional features* (PH-1) as the persistence of cycle structures. When thinking of these persistent intervals as bars, we can construct a *barcode* using the finite multi-set of bars to record the PH features, where PH-0 is represented using *β*_0_ barcode (Fig. 2b), PH-1 is using *β*_1_ barcode (Fig. 2c). Particularly, we can record the cycle structures over the graph filtration, and then integrate them to a *frequency network* which contains the specific connectivity information of the network.

Because there is no definite rule to determinate the proper *λ*_1_ and *λ*_2_ for the proposed model in Eqs. (2, 3), it will lead to inconsistency of network structure and uncertainty of results that follow. The problem can be remedied by using PH to perform statistical inference over every possible *λ*. More specifically, suppose that a group of brain networks *N*_G_ = (*N*_*λ*1_, *N*_*λ*2_, …, *N*_*λn*_) corresponding to different regularization parameters (*λ*_1_ < *λ*_2_ < , …, < *λ*_*n*_) rather than a fixed parameter, we can integrate the network group into an integrated network *N*_*int*_ (see Fig. 1b). The elements in *N*_*int*_ can be defined as probability-of-appearance of an edge in the network group *N*_*G*_. Assuming *γ* = 1 or 0 represents an edge exists or not, *n* is the number of networks in *N*_G_, we use distance network *N*_*d*_ to convert the elements of *N*_*int*_ by $d_{ij} = \sqrt {1 - \left( {\sum\nolimits_{l}^{n} {\gamma_{ij}^{l} } /n} \right)^{2} } \in N_{d}$ (see Fig. 1c). Then, a graph filtration for *N*_*d*_ can be constructed as follows: (1) Initial step is corresponding to the set of all brain regions; (2) Linearly increase the filtration distance *ε* (i.e., threshold) within the interval [0, 1], where the maximum number of generated networks is set as 1000; (3) For each *ε*, threshold the weighted distance network *N*_*d*_ using *d*_*ij*_ < *ε* to construct a binary network; (4) In the end, all brain nodes will be connected to one large unit. PH can be used to encode the graph filtration using the PH-0 and PH-1***.***

*0-dimensional features* (PH-0) We can obtain a *β*_0_ curve by fitting the *β*_0_ barcode using a *functional data analysis* (FDA) which can track the *β*_0_ curve features by combining *b*-spline basis functions with proper choice of roughness penalties [28, 29] (see Fig. 2b). Mathematically, the *β*_0_ curve function $y_{{\beta_{0} }}$ can be defined as:

4 $$y_{{\beta_{0} }} = \sum\nolimits_{k = 1}^{j} {c_{k} \varphi_{k} (t) + \varepsilon_{j} = {\varvec{c}}^{T} \user2{\varphi }(t) + } \varepsilon_{j}$$

where $\phi$ is an order four *b*-spline basis, and ***c*** contains the penalty coefficients, the residuals $\varepsilon_{j}$ is statistically independent. However, the fitting curves are not smooth because the process simply interpolates these points with lines. The problem can be addressed by minimizing the Eq. (5), which provides a compatible between capturing important curve features and reduce computations.

5 $$\min F(c) = \sum\nolimits_{j} {[y_{j} - c^{T} \varphi_{j} ]^{2} + \lambda \int {(c^{T} \varphi_{j} )^{2} dt} }$$

where the first term on the right side is the ordinary sum of squared errors of residuals, and the second term is the measure of roughness. The smoothing parameter *λ* specifies the emphasis on the integration relative to the goodness of fit in the SSE. As *λ* approaches positive infinity, curves become less rough and converge to a straight line. A theme in functional data analysis is the possibility of also using information on the rates of change or derivatives of the curves [30], because these curves are intrinsically smooth. The derivative curve of *β*_0_ fitting can magnify the curve’s features, thereby measuring the network difference through the curve distance measures like Cityblock and standardized Euclidean (Seuclidean) distance.

1*-dimensional features* (PH-1): A *persistence landscapes* method can convert the nonstandard and nonlinear 1-dimensional features to a sequence of piecewise-linear functions in Banach space, so we can use the linear vector space structures [31]. Their calculation is much faster than the corresponding barcode calculation. Given a ***β***_**1**_ barcode interval (*b*, *d*) with *b* < *d*, the piecewise linear function is defined as:

6 $$f_{(b,d)} (x) = \left\{ \begin{array}{ll} \, 0 &\quad if \, x \notin (b,d) \hfill \\ \, x - b &\quad if \, x \notin (b,\frac{b + d}{2}) \hfill \\ \, - x + b &\quad if \, x \notin (\frac{b + d}{2}{,}d) \hfill \\ \end{array} \right.$$

The persistence landscapes of a *β*_**1**_ barcodes $\left\{ {\left( {b_{i} ,d_{i} } \right)} \right\}_{i = 1}^{n}$ is a sequence of function *λ*_*k*_ so that *λ*_*k*_ is equals to the *k*-*th* largest value of $\left\{ {f_{{\left( {b_{i} ,d_{i} } \right)}} \left( x \right)} \right\}_{i = 1}^{n}$. More specifically, for every fixed *k*, *λ*_*k*_ can be regarded as exterior contours of a group of pairwise linear functions. Statistically, the great advantage of persistence landscapes is that, it is possible to compute the unique mean landscapes for a collection of persistent landscapes by taking the average for every landscape layer [32] (see Fig. 2c). This is not possible for barcodes, as they are not elements of a Banach space. The *L*_***p***_ distances can be used to quantify the difference between $\lambda_{k} (t)$ and $\lambda^{\prime}_{k} (t)$ corresponding to two persistent landscapes. In addition, we can not only calculate the *L*_***p***_ distance for $1 \le p \le \infty$ between the pairwise landscapes, but also between the average landscapes for two groups of persistence barcodes when analyze statistically. That allows one to compare multiple groups of *β*_**1**_ barcodes by calculating the pairwise similarity between them.

### Parameter selection

In the proposed multimodal modeling framework, there are two free parameters: *l*_1_-regularization parameter *λ*_1_, and fused Lasso parameter *λ*_2_, which control the performance of evaluation. A grid search is applied to search the optimal parameter combination. Of note, for obtaining the sequence of networks with different sparsity, a group of regularization parameter *λ*_1_ are selected in the range of [0, 0.9] with a uniform step size. Hence, we set the total sampling number of *λ*_1_ as a free parameter *N*_*λ*1_, and its candidate values for grid search are [100, 200, …, 500]. The candidate values for the fused Lasso parameter *λ*_2_ are [0.1, 0.2, …, 0.8]. In a word, our method involves the two parameters {*N*_*λ*1_, *λ*_2_} which should be optimized for receiving the most specific difference in APOE ε4-related group analysis.

### Statistical analysis and comparison

For the functional network, the structural network, and the proposed multimodal network, group-level significant differences between APOE ε4 carriers and non-carriers are computed (Fig. 1d). A non-parametric permutation test (see Additional file 1: Appendix S1) is used to assess the statistical difference for PH measurements (i.e., PH-0 and PH-1), while the graph theory measurements [33] such as local efficiency (LE), betweenness (BET), global efficiency (GE), and clustering coefficient (CCO) are evaluated using a two-sample t-tests. Significance is determined using a level of 0.05. For subject-level network differences, we denote *distances within group* (DWG) as the distances between all pairs of subjects within a group. For example, DWG of the APOE *ε*4 carriers with 27 subjects can be a vector composed of 351 pairwise distances. Similarly, the *distances between groups* (DBG) indicate the distances between all pairs of subjects from different groups. For instance, the DBG between APOE ε4 carriers (27 subjects) and non-carriers (36 subjects) could consist of 972 pairwise distances. In this study, Euclidean distance is used to measure the distances in DWG and DBG. Furthermore, we compared the PH metrics and the competing graph theory metrics.

### Connectivity pattern identification

We develop a connectivity pattern identification method for exploring the specific connectivity structures for APOE ε4 carriers and non-carriers, respectively. First, during the graph filtration, the brain regions synchronizing in a cycle structure could reflect a more meaningful neurobiological communication pattern in high dimensional space. Second, topological features with longer persistence could be assumed to convey important information about the brain network, while short ones are associated with noise. Hence, a *frequency network* which integrates the highest persistent cycles can encode the important brain connectomic information, and be used to identify the specific connectivity pattern for different APOE ε4 groups.

Suppose we have *n* frequency networks **Θ** for *n* APOE ε4 carriers, and *m* frequency networks **Φ** for *m* APOE ε4 non-carriers in Eq. (7). After adding the weights of every corresponding edges together for each frequency network group, we can achieve the network ${\tilde{\mathbf{\Theta }}}$ or ${\tilde{\mathbf{\Theta }}}$ in Eq. (8). A difference analysis is performed for each specific connectivity. Subsequently, we select a threshold (*ε*_*t*_ = 2) to filter the difference results and to obtain the ensuing *difference network* (binary network) ***Δ***_ε4_ or ***Δ***_*non-*ε4_ in Eq. 9. That is to say, the specific edges in network ***Δ***_ε4_ could appear more often in the APOE ε4 carriers than non-carriers. Furthermore, compared to the edge measurement in ***Δ***_ε4_ or ***Δ***_*non-*ε4_, the specific loops could reflect the potential biological communication pattern. In this study, we further extract the loop structures from ***Δ***_ε4_ or ***Δ***_*non-*ε4_ to quantify the APOE ε4-associated connectivity.

7 $${{\varvec{\Theta}}} = \{ \Theta_{1} ,\Theta_{2} , \ldots ,\Theta_{n} \} ,\quad {{\varvec{\Phi}}} = \{ \Phi_{1} ,\Phi_{2} , \ldots ,\Phi_{m} \}$$

8 $${\tilde{\mathbf{\Theta }}} = \sum\nolimits_{i = 1}^{n} {\Theta_{i} } ,\quad {\tilde{\mathbf{\Phi }}} = \sum\nolimits_{j = 1}^{m} {\Phi_{j} }$$

9 $$\Delta_{\varepsilon 4} = {\tilde{\mathbf{\Theta }}} - {\tilde{\mathbf{\Phi }}} < \varepsilon_{t} ,\quad \Delta_{non - \varepsilon 4} = {\tilde{\mathbf{\Phi }}} - {\tilde{\mathbf{\Theta }}} < \varepsilon_{t} .$$

## Results

### Performance comparison

**Fig. 3:**
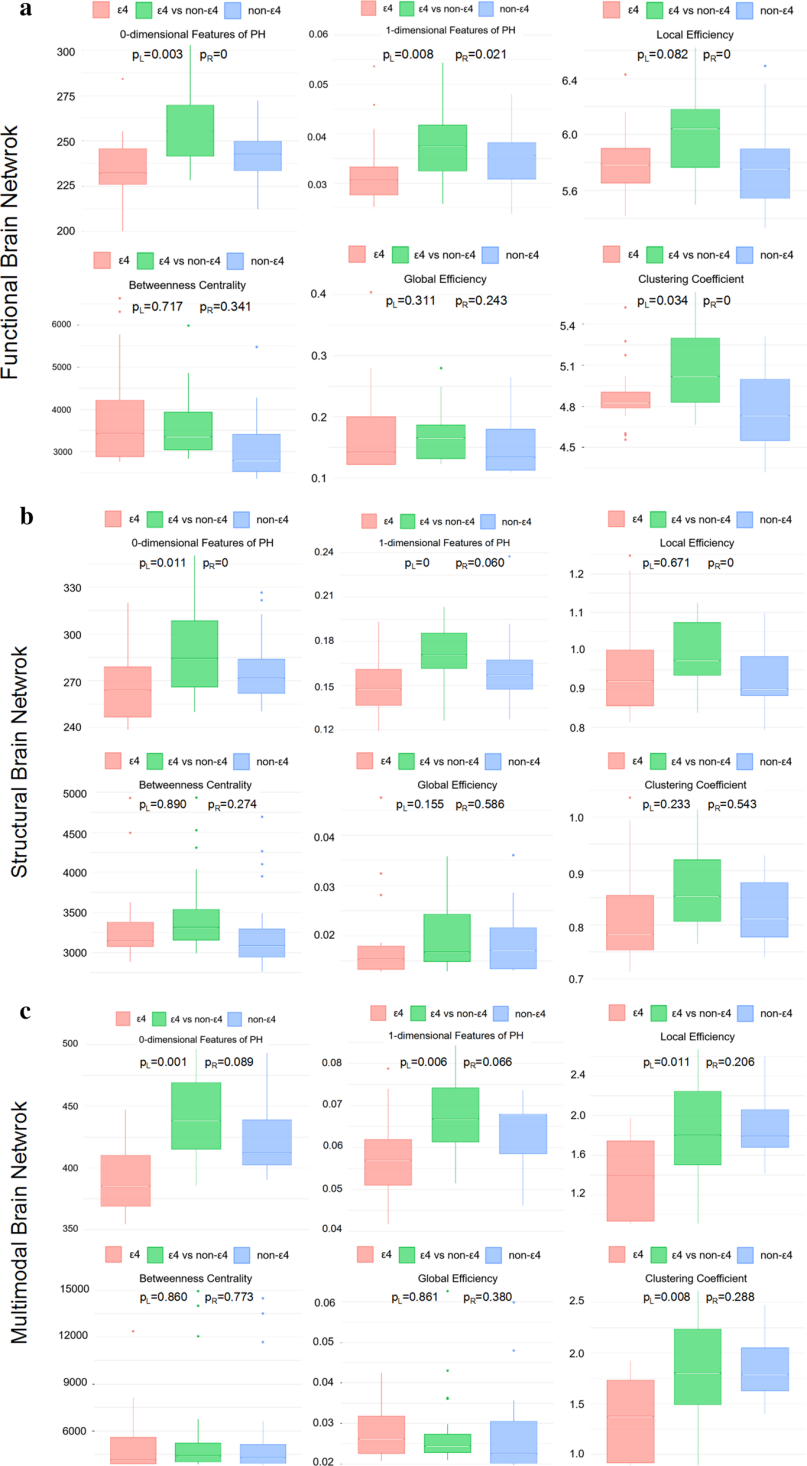
For **a** the functional connectivity, **b** the structural connectivity, and **c** the proposed multimodal brain connectivity, the distance between groups (DBG, in green) and the distance within group (DWG, ε4 group in red and non-ε4 group in blue) are given. We compared the persistent homology features and some graph theory metrics. A discriminative feature corresponds to a large DBG (green) against two small DWGs (red and blue). In addition, p_L_ and p_R_ represent the statistical significance using t-test between any columns, i.e. DWG (ε4) and DBG, DWG (non-ε4) and DBG, respectively

Table 1 shows the significance (*p*-value of two-sample t-test) of group comparison results (APOE ε4 carriers vs non-carriers) using six network measures from three types of brain networks. From Table 1, no statistically significant differences (*p* > 0.05) were found in terms of LE, BET, GE, CCO, and PH-1, of the whole brain FC. But a significant difference in PH-0 were observed, *p* = 0.0244 < 0.05, between APOE ε4 carriers and non-carriers. For the whole brain SC, one significant difference in PH-0 was found, *p* = 0.0081 < 0.05, and the groups do not differ significantly in other measurements, which is similar to FC. Of note, for the SC, *p* values is lower than that in FC, which represents a more significant difference shown in SC. Especially, PH-1 shows a significant improvement relative to that in FC, and the result is marginally significant with *p* = 0.056. For the proposed multimodal brain network, our method outperforms the competing FC and SC. The statistically significant differences were found in LE (*p* = 0.0196), CCO (*p* = 0.0178), PH-0 (*p* = 0.0001), and PH-1 (*p* = 0.0321) between groups. We further quantified the subject-wise network differences using DWG and DBG for the FC, the SC, and the multimodal network (see Fig. 3) and performed a paired t-test between neighbor columns in each boxplot. We found that the PH metrics reflect the difference of networks better than graph metrics like LE, BET, GE, and CCO. That is to say, the DBG (green) between APOE ε4 carriers and non-carriers should be higher than the DWG within carriers (red) or DWG within non-carriers (blue).

**Table 1:** The significance (*p*-value of a two-sample t-test) of group comparison results (APOE ε4 carriers vs non-carriers) using six network measures from three types of brain networks

	LE	BET	GE	CCO	PH-0	PH-1
FC	0.4388	0.6570	0.9791	0.5022	0.0244	0.1562
SC	0.0947	0.4632	0.1927	0.0526	0.0081	0.0560
Proposed	0.0196	0.1469	0.2756	0.0178	0.0001	0.0321

The three types of brain networks include functional connectivity networks (FC), structural connectivity networks (SC), and the networks constructed using our proposed method (Proposed). The six network measures include local efficiency (LE), betweenness (BET), global efficiency (GE), clustering coefficient (CCO), and two PH measurements (PH-0 and PH-1)

### The influence of regularizations

**Fig. 4:**
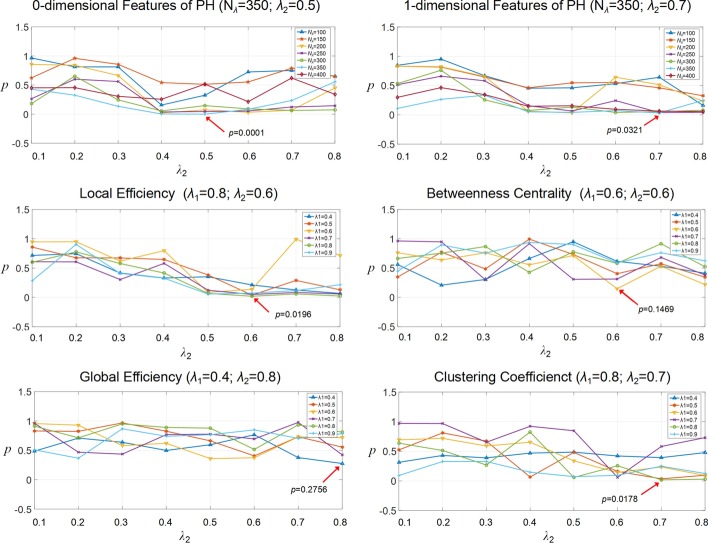
The statistical performance of different regularization parameters. For 0-dimensional features and 1-dimensional features, the candidate parameters for grid search are sampling number *N*_*λ*1_ and regularization parameter *λ*_2_. For other graph theory measurements, the candidate parameters are regularization parameter *λ*_1_ and *λ*_2_

We applied grid search to explore the influence of regularizations for statistical group analysis. From Fig. 4, the performances in LE, BET, GE, and CCO are irregular, which is because graph theory measurements have a strong parametric sensitivity for network structures. In addition, we found that when *N*_*λ*_ is small (*N*_*λ*_ = 100, 150, and 200), *p* values in PH-0 remain a relatively high level (lower significance level). With an increasing value of *N*_*λ*_, *p* value achieves the best performance (*p* = 0.001) at *N*_*λ*_ = 350, and then shows a suboptimal performance again at *N*_*λ*_ = 400. For PH-1, the trend is almost similar to PH-1 that the best performance is received (*p* = 0.0321) at *N*_*λ*_ = 350. For *λ*_2_, we found *p* value shows a growth trend firstly, and after achieving the optimization *λ*_2_ (0.5 and 0.7 for PH-0 and PH-1), the performance tends to decrease.

### Connectivity pattern identification

**Fig. 5:**
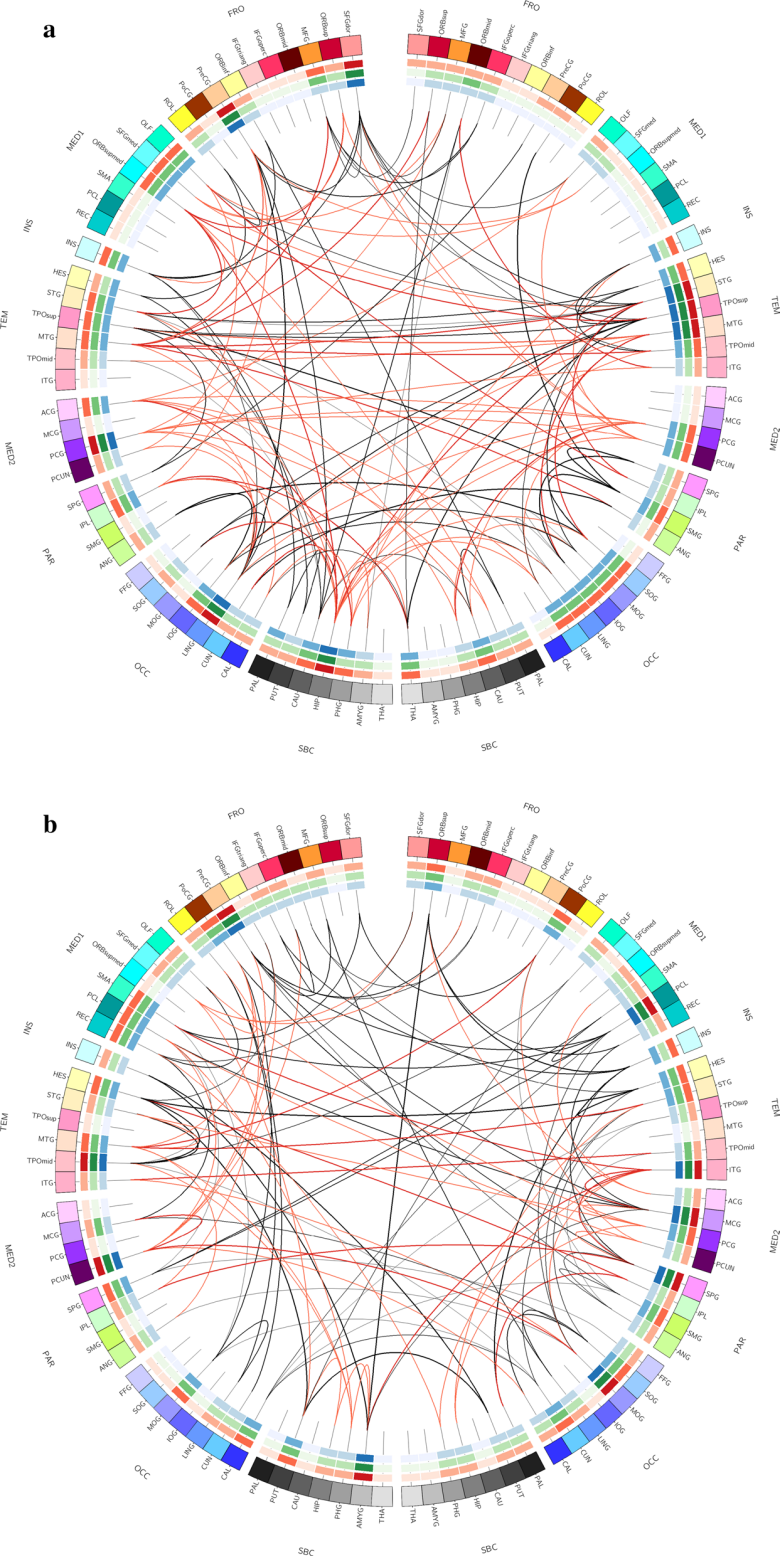
The specific connectivity for **a** APOE ε4 carriers, **b** APOE ε4 non-carriers, where red arcs represent the DMN-associated connections and black arcs extend the connections to whole brain

After obtaining the frequency network for every subject, we constructed the difference network between groups by the proposed connectivity pattern identification framework. Furthermore, we extracted the loops from each difference network corresponding to APOE ε4 group or non-ε4 group, where it totally has 40 loops for ***Δ***_ε4_ and 35 loops for ***Δ***_*non-*ε4_. In particular, we defined the number of edges forming the loops as a weight, and then selected the top 8 loops with the largest weights (see Additional file 1: Appendix S2). It is worth mentioning that the loop structures might not entirely appear in the brain network of each subject, but in just parts of them. Figure 5 graphically illustrates that the extracted 8 loops for APOE ε4 carriers and non-carriers, respectively, where the red arcs represent the connections associated with the default mode network (DMN) that has been commonly regarded as AD-pathology related. The black arcs in Fig. 5 denote the connections outside the DMN, which extends the previous studies of DMN to the whole brain level.

## Discussion

### Efficacy of the multimodal modelling

In this paper, we compared the rs-fMRI networks, the dw-MRI networks, and the multimodal networks. We found that there were no significant differences between APOE ε4 carriers and non-carriers in the graph measurements of the rs-fMRI and dw-MRI networks. But compared with the rs-fMRI networks, a relatively big difference was observed in the dw-MRI networks. This finding is consistent with prior studies [6, 9, 34], which found that APOE *ε*4 carriers show relatively poorer SC than FC, involving disrupted white matter microstructural organization, smaller brain volumes, and lower regional SC in DMN. Furthermore, when integrating multimodal information from the rs-fMRI and dw-MRI data using our proposed framework, we observed a significant group difference in LE, CCO, PH-0, and PH-1. Although the same significant difference is observed in PH-0 for the FC, the SC, and the multimodal network, the proposed multimodal network showed the best performance (*p* = 0.0001). Our observation indicates that the multimodal network is more sensitive than either rs-fMRI or dw-MRI alone in exploring specific connectivity properties related to risk of Alzheimer's disease. This result demonstrates the advantage of multimodal integration using the dw-MRI connectivity to guide the fMRI-based network construction.

### Efficacy of persistent homology

We proposed a novel framework for quantifying network features using PH. The proposed framework integrates a group of brain networks with different sparsity levels, which avoids the uncertainty of regularization parameter selection. Moreover, instead of trying to obtain an unbiased estimate of connectivity strength, our method focused on the binary network structure rather than the weight-based connectivity network. Statistically, we found persistent homology features show more significant difference than other graph theory measurements at the group level. Especially, the PH-0 outperforms the PH-1 in statistical performance, which represents the connected components is more sensitive in encoding graph filtration than the topological cycle structures. After evaluating the subject-level difference using DWG and DBG, persistent homology features showed a better discriminating power than others. It is also worth noting that the DWG of APOE ε4 non-carriers should be larger than the DWG of the APOE ε4 carriers, i.e., the blue boxplot in Fig. 3 is typically higher than the red one. This indicates that the carrier group appears more homogeneous than non-carrier group. Furthermore, the above patterns were detected only by the multimodal network with PH quantification, but not by any other studied methods. A similar approach fusing persistent homology and sparse representation has been used by [14] for characterizing the abnormality of white matter network, but they investigated a sparse version of pair-wise ROI correlation, rather than multiple co-activated ROIs, and explore the single modality rather than multiple modalities.

### Efficacy of regularizations

Brain networks corresponding to various combinations of *l*_1_-regularization parameter *λ*_1_ and fused Lasso parameter *λ*_2_ have different sparsity levels, leading to a variety of different results. The bigger the *l*_1_-norm regularization parameter, the lower nonzero ratio will be obtained for brain network. Different from *l*_1_-norm regularization parameter, the fused Lasso parameter handles the feature collinearity for improved stability. By grid search to adjust the two parameters, the best combination of parameters can improve the results of statistical analysis. This demonstrates the effectiveness of searching the parameters to characterize the APOE ε4-related network difference.

### The evaluation of specific connectivity

The significant decrease in brain connectivity caused by AD could modularize regional brain atrophy. On the other hand, brain regions involved in a topological cycle with long persistence are potentially located in relatively independent modules in the brain network. The ***frequency network*** aims to record the frequent edges in a group of cycles, because the frequent edges could connect to the atrophied brain regions in APOE *ε*4 carriers with a higher probability. The ***difference network*** specializes these frequent edges. We further extracted the specific loops structures from the difference network as the results of connectivity pattern identification. After evaluating these loop structures, we found brain regions in APOE *ε*4 carriers mostly refer to the interconnection within the DMN than non-carriers. Especially, we found that compared with non-carriers, APOE *ε*4 carriers exhibit one cluster involving the connections between left temporal gyrus and right temporal gyrus, mainly focusing on left middle temporal gyrus, left superior temporal gyrus, right superior temporal gyrus, right middle temporal gyrus, right inferior temporal gyrus. Moreover, APOE *ε*4 carriers show the specific connectivity within a sub-network centering on the left hippocampus, extending into left/right cuneus, left/right superior occipital gyrus, right precuneus, left Inferior parietal lobule, left middle temporal gyrus, right temporal pole middle gyrus, right superior temporal gyrus, right thalamus, and left superior frontal gyrus (medial orbital). These results were founded to be associated with brain network of APOE *ε*4 carriers in previous researches [6, 8, 35], and further indicate the effectiveness of the proposed connectivity pattern identification method.

### Limitation and future directions

Of note, in this study, there are two limitations needing to be improved next for the proposed method. First, our method could identify cycles consisting of the greatly changed brain regions, rather than mildly changed regions. Because significant edges between regions as the modulator have a longer persistent interval in graph filtration, which can be easy to observe using PH. This limitation may potentially influence the result of connectivity pattern identification. Second, a relatively small number of subjects remains a problem for statistical analysis. In the future, we will improve the performance of structure identification using a more specific brain partition and validate our framework on a larger dataset.

## Conclusions

In this study, we have proposed a novel multimodal brain network modeling framework for identifying the APOE ε4-related differences in the brain connectome. To integrate brain network information of different sparsity levels as well as avoid extra connectivity strength estimation, we have introduced persistent homology (PH) to quantify the individual network. Experimental results on the ADNI database demonstrated that the proposed framework could generate multimodal brain networks with great discriminative power. Moreover, the persistent homology features outperformed other measurements when quantifying APOE ε4-related network differences. In addition, the specific connectivity pattern could be obtained by encoding the 1-dimensional features of PH. These specific structures were consistent with previous results in DMN and expanded DMN to whole brain connectivity. These findings suggest that the proposed method not only improves the statistical performance between APOE ε4 carriers and non-carriers, but also characterizes the interaction effects between brain connectivity and APOE ε4.

## Supplementary information

**Additional file 1.** The permutation test workflow and the identified disease-specific connectivity pattern.

## Data Availability

Data used in the preparation of this article were obtained from the ADNI database (adni.loni.usc.edu). The ADNI was launched in 2003 as a public–private partnership, led by Principal Investigator Michael W. Weiner, MD. The primary goal of ADNI has been to test whether serial magnetic resonance imaging (MRI), positron emission tomography (PET), other biological markers, and clinical and neuropsychological assessment can be combined to measure the progression of MCI and early AD.

## Abbreviations

AD: Alzheimer’s disease
APOE: Apolipoprotein E
rs-fMRI: Resting state functional magnetic resonance imaging
dw-MRI: Diffusion-weighted magnetic resonance imaging
DMN: Default mode network
Lasso: Least absolute shrinkage and selection operator
PH: Persistent homology
SC: Structural connectivity
FC: Functional connectivity
TE: Echo time
TR: Repetition time
FA: Filp angle
PH-0: 0-Dimensional features of persistent homology
PH-1: 1-Dimensional features of persistent homology
LE: Local efficiency
BET: Betweenness
GE: Global efficiency
CCO: Clustering coefficiency
DWG: Distances within group
DBG: Distances between groups

## References

[1] Klaassens BL (2019). Cholinergic and serotonergic modulation of resting state functional brain connectivity in Alzheimer's disease. Neuroimage.

[2] Agosta F (2012). Resting state fMRI in Alzheimer's disease: beyond the default mode network. Neurobiol Aging.

[3] Chang YT (2019). APOE-MS4A genetic interactions are associated with executive dysfunction and network abnormality in clinically mild Alzheimer's disease. NeuroImage Clin.

[4] Bussy A, Snider BJ, Coble D (2019). Effect of Apolipoprotein E4 on clinical, neuroimaging and biomarker measures in non-carrier participants in the Dominantly Inherited Alzheimer Network. Neurobiol Aging.

[5] Andrews JR (2010). Functional-anatomic fractionation of the Brain's default network. Neuron.

[6] Korthauer LE, Zhan L, Ajilore O (2018). Disrupted topology of the resting state structural connectome in middle-aged, APOE, ε4 carriers. Neuroimage.

[7] Shen L, Thompson PM (2020). Brain imaging genomics: integrated analysis and machine learning. Proc IEEE..

[8] Patrizia AC (2019). Differential default mode network trajectories in asymptomatic individuals at risk for Alzheimer's disease. Alzheimer’s Dement.

[9] Pietzuch (2019). The influence of genetic factors and cognitive reserve on structural and functional resting-state brain networks in aging and Alzheimer's disease. Front Aging Neurosci.

[10] Dawei W (2020). KIBRA and APOE gene variants affect brain functional network connectivity in healthy older people. J Gerontolo Ser A.

[11] Cai B, Zille P, Stephen JM (2018). Estimation of dynamic sparse connectivity patterns from resting state fMRI. IEEE Trans Med Imaging.

[12] Yu RP, Qiao LS (2019). Weighted graph regularized sparse brain network construction for MCI identification. Pattern Recogn.

[13] Peng C, Xiaoli L, Hezi L (2018). Generalized fused group Lasso regularized multi-task feature learning for predicting cognitive outcomes in Alzheimers disease. Comput Methods Programs Biomed.

[14] Chung MK, Hanson JL, Ye J, Davidson RJ, Pollak SD (2015). Persistent homology in sparse regression and its application to brain morphometry. IEEE Trans Med Imag.

[15] Li Y (2019). Novel effective connectivity inference using ultra-group constrained orthogonal forward regression and elastic multilayer perceptron classifier for MCI identification. IEEE Trans Med Imaging.

[16] Bubenik P, Kim PT (2007). A statistical approach to persistent homology. Homology Homotopy and Applications.

[17] Stolz BJ (2017). Persistent homology of time-dependent functional networks constructed from coupled time series. Chaos.

[18] Kuang LQ (2019). A concise and persistent feature to study brain resting-state network dynamics: Findings from the Alzheimer's Disease Neuroimaging Initiative. Hum Brain Mapp.

[19] Lee H (2012). Persistent brain network homology from the perspective of dendrogram. IEEE Trans Med Imaging.

[20] Zhang Y (2019). Strength and similarity guided group-level brain functional network construction for MCI diagnosis. Pattern Recogn.

[21] Li Y, Liu J (2019). Multimodal hyper-connectivity of functional networks using functionally-weighted LASSO for MCI classification. Med Image Anal.

[22] Pinedapardo J, Bruña R (2014). Guiding functional connectivity estimation by structural connectivity in MEG: an application to discrimination of conditions of mild cognitive impairment. Neuroimage.

[23] Qi S (2018). Multimodal fusion with reference: searching for joint neuromarkers of working memory deficits in schizophrenia. IEEE Trans Med Imaging.

[24] Yan CG (2016). DPABI: data processing and analysis for (resting-state) brain imaging. Neuroinformatics.

[25] Cui ZX (2013). PANDA: a pipeline toolbox for analyzing brain diffusion images. Front Hum Neurosci.

[26] Zomorodian A, Carlsson G (2005). Computing persistent homology. Discrete Comput Geom.

[27] Xia KL (2018). Persistent homology analysis of ion aggregations and hydrogen-bonding networks. Phys Chem Chem Phys.

[28] Ramsay and Silverman (2005). Functional data analysis.

[29] Graves S (2009). Functional data analysis.

[30] Cassidy B (2018). On the reliability of individual brain activity networks. IEEE Trans Med Imaging.

[31] Bubenik P (2015). Statistical topological data analysis using persistence landscapes. J Mach Learn Res.

[32] Bubenik P, Dlotko P (2017). A persistence landscapes toolbox for topological statistics. J Symb Comput.

[33] Bullmore ET, Sporns O (2009). Complex brain networks: graph theoretical analysis of structural and functional systems. Nat Rev Neurosci.

[34] Zhangjia D (2019). Disrupted structural and functional brain networks in Alzheimer’s disease. Neurobiol Aging.

[35] Luo X, Li K (2018). Altered effective connectivity anchored in the posterior cingulate cortex and the medial prefrontal cortex in cognitively intact elderly APOE ε4 carriers: a preliminary study. Brain Imaging Behav.

