# Supplementary material for: Effect of APOE ε4 on multimodal brain connectomic traits: a persistent homology study
Source: BMC Bioinformatics. 2020 Dec 28;21(Suppl 21):535. doi: 10.1186/s12859-020-03877-9 (PMC7768655; doi:10.1186/s12859-020-03877-9)
Supplement: Supplementary file 1 — Additional file 1. The permutation test workflow and the identified disease-specific connectivity pattern. [file 12859_2020_3877_MOESM1_ESM.pdf]

Supplementary Materials for "Effect of APOE ε4 on Multimodal Brain Connectomic Traits: A Persistent Homology Study" by Jin Li, Chenyuan Bian, Dandan Chen, Xianglian Meng, Haoran Luo, Hong Liang, Li Shen, for the Alzheimer's Disease Neuroimaging Initiative

Appendix S1. A permutation test is used to evaluate the group difference statistically for APOE ε4 carriers and non-carriers using the multi-dimensional persistent features, where the flow of (1)—(2)—(3a)—(4)—(5)—(6) is corresponding to 0-dimensional features, and the flow of (1)—(2)—(3b)—(4)—(5)—(6) is for 1-dimensional features.

Permutation test for multi-dimensional features

---

(1) Pairwise statistical comparisons for the two groups $G_{\varepsilon 4}$, $G_{non\text{-}\varepsilon 4}$. The null hypothesis is $\tilde{G}_{\varepsilon 4} = \tilde{G}_{non-\varepsilon 4}$.

(2) **a) For 0-dimensional features**: Given $\tilde{G}_a = \{X_1, \dots, X_N\}_{n\times N}$, $\tilde{G}_b = \{Y_1, \dots, Y_M\}_{n\times M}$, where N and M are the numbers of subjects in two groups. X and Y are the derivative curve vectors with uniform sampling points. We define $\mathbb{T}$ in the following equation as the statistics and then calculate the statistics $\mathbb{T}_0$ of the null hypothesis. Permute the subjects in $\tilde{G}_a$ and $\tilde{G}_b$ by grouping randomly to construct two pseudo-collections.

$$\mathbb{T} = \text{mean}(\left|(\overline{\tilde{G}_a})_{n\times 1} - (\overline{\tilde{G}_b})_{n\times 1}\right|)$$

b) For 1-dimensional features: $\tilde{G}_a = \{\boldsymbol{\lambda_1}, \dots, \boldsymbol{\lambda_N}\}$, $\tilde{G}_b = \{\boldsymbol{\lambda'_1}, \dots, \boldsymbol{\lambda'_M}\}$, where $\boldsymbol{\lambda}$ and $\boldsymbol{\lambda'}$ are the persistence landscapes for each subject in $\tilde{G}_a$ and $\tilde{G}_b$. After calculating the average landscapes for each group in the following equation, the $\mathbb{L}_p$ (p=1) distance between $\bar{\lambda}_a$ and $\bar{\lambda}'_b$ can be regarded as the statistics. Therefore, the null hypothesis is corresponding to $\mathbb{L}_0$. Permute the elements of $\tilde{G}_a$ and $\tilde{G}_b$ and construct two pseudo-collections.

$$\bar{\lambda}_a = \sum_{j=1}^{N} \frac{1}{N} \lambda_j,\ \bar{\lambda}'_b = \sum_{i=1}^{M} \frac{1}{M} \lambda'_i$$

(3) The permutation is executed 10,000 times. We achieve the statistics order $\{\mathbb{T}'_1, \dots, \mathbb{T}'_{10^4}\}$ or $\{\mathbb{L}'_1, \dots, \mathbb{L}'_{10^4}\}$ as a permutation distribution. The p-value can be calculated as the proportion of sampled permutations by measuring the number of elements greater than $\mathbb{T}_0$ or $\mathbb{L}_0$ in the statistics order.

(4) According to the predefined confidence interval (0.05), we estimate the statistics significance by the p-value.

Appendix S2. The results of connectivity pattern identification, including the specific loop structures for APOE ε4 carriers and non-carriers respectively. The number in parentheses represents the index of 116 AAL brain regions.

Name	Specificity loops

ε4	—PoCG.R(58)—SMG.R(64)—TPOsup.L(83)—MTG.R(86)—IOG.R(54)—SPG.R(60)—SMG.R(64)—SOG.R(50)—ANG.L(65)—IOG.L(53)—TPOsup.R(84)—THA.R (78)—IPL.L(61)—LING.L(47)—PreCG.L(1)—MTG.L(85)—ANG.R(66)—CUN.L(45)—HIP.L(37)—SOG.L(49)—
ε4	—OLF.R(22)—ACG.L(31)—CAU.R(72)—PAL.R(76)—ORBinf.L(15)—INS.L(29)—PAL.L(75)—PUT.L(73)—HES.L(79)—ROL.L(17)—OLF.L(21)—PCUN.L (67)—LING.L(47)—LING.R(48)—PHG.L (39)—INS.R (30)—PCUN.R (68)—SFGdor.L (3)—
ε4	—PreCG.R(2)—SPG.L(59)—TPOsup.L(83)—TPOsup.R(84)—HES.R(80)—STG.L(81)—ORBsupmed.L(25)—INS.L(29)—SFGdor.L(3)—STG.R(82)—SFGmed.L(23)—ORBsup.L(5)—PCUN.R (68)—HIP.L(37)—IPL.L(61)—PreCG.L (1)—CAU.L (71)—ORBmid.R(10)—
ε4	—TPOsup.L(83)—MTG.R(86)—TPOmid.L(87)—CAU.L (71)—PCG.R(36)—HIP.R(38)—MOG.R(52)—IOG.R(54)—LING.R(48)—STG.L(81)—STG.R(82)—MTG.L(85)—HIP.L(37)—AMYG.L(41)—ORBmid.R(10)—ITG.R(90)—PCG.L(35)—MFG.R(8)—
ε4	—PreCG.R(2)—SPG.L(59)—TPOsup.L(83)—STG.R(82)—HES.R(80)—STG.L(81)—CAU.L(71)—CAU.R(72)—ORBsup.L(5)—SFGmed.L(23)—MFG.R(8)—SFGdor.L(3)—OLF.R(22)—PCUN.R (68)—PCG.L(35)—PUT.R(74)—ORBsupmed.L(25)—
ε4	—SFGdor.R(4)—MFG.L(7)—SFGdor.L (3)—TPOmid.R(88)—HIP.L(37)—STG.R(82)—OLF.L(21)—SFGmed.R(24)—PreCG.L(1)—MTG.L(85)—ORBsup.R(6)—THA.R(78)—CAL.L(43)—PHG.L(39)—PCG.L(35)—PCG.R(36)—CUN.R(46)—ACG.L(31)—LING.L(47)—INS.L(29)—
ε4	—LING.L(47)—SOG.L(49)—TPOsup.R(84)—INS.R(30)—TPOmid.R(88)—MTG.L(85)—MFG.L(7)—IFGoperc.R(12)—PreCG.L(1)—SFGdor.L (3)—STG.R(82)—MOG.R(52)—IOG.L(53)—MTG.R(86)—SOG.R(50)—HIP.L(37)—THA.R (78) —
ε4	—SFGdor.R(4)—MFG.L(7)—TPOsup.R(84)—ORBsupmed.L(25)—HIP.L(37)—CUN.R(46)—STG.R(82)—IPL.R(62)—OLF.L(21)—SFGmed.R(24)—PreCG.L(1)—MTG.L(85)—ORBsup.R(6)—THA.R(78)—CAL.L(43)—PCG.L(35)—PHG.L(39)—ACG.L(31)—PCG.R(36)—LING.L(47)—
non-ε4	—STG.L(81)—TPOmid.L(87)—HES.L(79)—PUT.L(73)—PUT.R(74)—INS.L(29)—AMYG.L(41)—ORBsup.R(6)—INS.R(30)—PAL.R(76)—IPL.R(62)—HES.L(79)—HES.R(80)—STG.R(82)—
non-ε4	—PreCG.L(1)—MFG.L(7)—LING.R(48)—SOG.R(50)—CAL.R(44)—PCUN.L(67)—IFGoperc.L(11)—IFGtriang.L(13)—INS.R(30)—SPG.R(60)—PoCG.L(57)—IPL.L(61)—PoCG.R(58)—MOG.L(51)—MOG.R(52)—LING.L(47)—ROL.L(17)—CUN.L(45)—
non-ε4	—SPG.R(60)—PCUN.R(68)—PreCG.R(2)—SFGdor.L(3)—SMA.L(19)—PCL.R(70)—SPG.L(59)—SMA.R(20)—PCL.R(70)—PreCG.L(1)—MCG.R(34)—PCL.L(69)—PHG.L(39)—AMYG.L(41)—HIP.L(37)—PUT.L(73)—
non-ε4	—FFG.R(56)—SPG.L(59)—PCL.L(69)—TPOmid.L(87)—CUN.R(46)—MOG.L(51)—SMA.L(19)—ITG.R(90)—SPG.R(60)—TPOmid.R(88)—ITG.L(89)—MOG.R(52)—ANG.L(65)—MTG.L(85)—PoCG.R(58)—SMG.R(64)—CAL.L(43)—TPOsup.R(84)—ANG.R(66)—
non-ε4	—PCUN.R(68)—PCL.L(69)—PCUN.L(67)—MCG.L(33)—SOG.R(50)—STG.R(82)—CUN.R(46)—CAL.L(43)—PCL.R(73)—MOG.R(52)—ITG.R(90)—DCG.R(34)—PreCG.L(1)—IPL.L(61)—PoCG.R(58)—SPG.R(60)—ROL.L(17)—IPL.R(62)—SMG.R(64)—
non-ε4	—STG.L(81)—MTG.L(85)—PoCG.L(57)—IPL.L(61)—SPG.R(60)—ANG.R(66)—SMG.R(64)—PreCG.L(1)—IFGoperc.R(12)—ORBmid.L(9)—HES.R(80)—MOG.R(52)—PCUN.L(67)—ORBinf.L(15)—HES.L(79)—
non-ε4	—ORBsupmed.R(26)—ACG.R(32)—ORBsup.R(6)—REC.R(28)—MCG.R(34)—PCG.R(36)—OLF.L(21)—REC.L(27)—DCG.L(33)—PCUN.L(67)—PreCG.L(1)—ORBmid.R(10)—SFGmed.L(23)—ROL.L(17)—
non-ε4	—STG.L(81)—PCG.R(36)—HIP.R(38)—STG.R(82)—MTG.L(85)—PHG.L(39)—TPOmid.L(87)—OLF.R(22)—PUT.L(73)—SFGmed.L(23)—SFGdor.R(4)—REC.L(27)—PHG.R(40)—ITG.R(90)—AMYG.L(41)—DCG.R(34)—
